# Acute Effects of Brief Mindfulness Intervention Coupled with Carbohydrate Ingestion to Re-Energize Soccer Players: A Randomized Crossover Trial

**DOI:** 10.3390/ijerph17239037

**Published:** 2020-12-04

**Authors:** Yuxin Zhu, Fenghua Sun, Chunxiao Li, Daniel Hung Kay Chow

**Affiliations:** 1 Department of Health and Physical Education, The Education University of Hong Kong, Hong Kong, China; im.usinzu@gmail.com (Y.Z.); danielchow@eduhk.hk (D.H.K.C.); 2 School of Physical Education & Sports Science, South China Normal University, Guangzhou 510631, China; cxlilee@gmail.com

**Keywords:** recovery, team sport, fatigue

## Abstract

*Background:* This field experiment investigated the acute effects of brief mindfulness-based intervention (MBI) coupled with carbohydrate (CHO) intake on players’ recovery from half-time break in a simulated soccer competition. *Methods:* In a single-blinded randomized crossover experiment, 14 male players received 3 treatments (Control: non-carbohydrate solution + travelling introduction audio; CHO: CHO–electrolyte solution + travelling introduction audio; and CHO_M: CHO–electrolyte solution + MBI) during simulated half-time breaks. Vertical jump, sprint performance, mindfulness level, rate of perceived exertion, muscle pain, mental fatigue, blood glucose, and lactate were measured immediately before, during, and after the exercise. *Results:* (1) MBI significantly increased participants’ mindfulness level (Control vs. CHO_M, *p* < 0.01; CHO vs. CHO_M, *p* < 0.01) and decreased mental fatigue for CHO_M condition (pre vs. post, *p* < 0.01); (2) participants in the CHO_M condition performed better in the repeated sprint tests than in the Control and CHO condition (Control vs. CHO_M, *p* = 0.02; CHO vs. CHO_M, *p* = 0.02). *Conclusion:* Findings of this study provide preliminary evidence of the positive effect of MBI coupled with CHO ingestion on athletes’ recovery from fatigue in the early stage of the second half of a game.

## 1. Introduction

The game of soccer involves high-intensity intermittent exercise that can cause physiological and mental fatigue [1]. Physiological fatigue is generally defined as maximal voluntary muscle force reduction caused by exercise, while mental fatigue refers to a psychobiological status involving feelings of tiredness and lack of energy [1]. Laboratory-based research has demonstrated that both types of fatigue could adversely affect athletic performance. For example, muscle damage, resulting in physiological fatigue, impairs an athlete’s performance by increasing muscle pain and decreasing muscle force [2]. Meanwhile, research has shown that mental fatigue causes altered attentional focus and reduces the speed and preciseness of responses [3]. Thus, fast recovery from fatigue is crucial to an athlete’s performance. In soccer, the half-time break has been regarded as a good opportunity to recover from fatigue [4]. Therefore, it is necessary to investigate whether any strategies could facilitate soccer players’ recovery from physiological and mental fatigue during that critical period.

Fluid intake in the form of common sports drinks is one of the most popular recovery strategies, as it prevents excessive dehydration and provides various nutrients for recovery. Nutrients with fluids, such as carbohydrate (CHO), have been found to be important for both anaerobic and aerobic energy systems [5]. Research suggests that CHO ingestion during a soccer match maintains the blood glucose concentration and spares muscle glycogen, which may improve performance in soccer [6].

Although numerous studies have investigated the effects of fluid intake on recovery from physiological fatigue, little attention has been paid to understand the recovery of mental fatigue. Recently, the potential benefits of mindfulness have been researched widely by scholars in a diverse number of fields, including psychology, education, healthcare, and sports (e.g., athletes’ sleep [7,8] and burnout [9]). Mindfulness is defined as the awareness that emerges from paying attention to objects on purpose and without judging the unfolding of experience moment by moment [10]. The mindfulness-based intervention (MBI) programs that typically last for 8–12 weeks have been shown to reduce stress, anxiety, burnout, and pain [11]. In addition, brief MBI (i.e., 5–20 min/session) programs have been found to reduce chronic pain or increase pain tolerance [12,13], benefit cardiovascular and respiratory modulation [14], and help physiological relaxation responses [15].

To date, MBI has not been studied as a recovery tool in the context of sports competition. Considering the effectiveness of MBI in improving psychological outcomes [12,13,14,15,16,17], it is highly possible that MBI may provide beneficial effects in fatigue recovery after a 45 min soccer match. The present study therefore investigated the effect of brief MBI as an adjunct to CHO fluid intake to reenergize soccer players during half-time breaks. We hypothesized that the combination strategy would yield the best effect on recovery when compared with the other two conditions, i.e., CHO fluid intake with control audio and non-CHO fluid intake with control audio, respectively.

## 2. Materials and Methods

### 2.1. Participants

In total, 18 male soccer players were recruited, of which 14 completed the whole experiment (age: 24.3 ± 3.7 year, height: 1.74 ± 0.05 cm, weight: 68.3 ± 5.1 kg, VO_2max_: 47.0 ± 4.4 mL/kg/min; average training years: 2.5 years; drop-out rate: 22%). Participants reported no history of MBI practice. The present research was approved by the University Human Research Ethics Committee (Ref. no. 2018-2019-0221). All the participants gave their written consent prior to joining the experiment.

### 2.2. Experimental Protocol

A 3-treatment, single-blinded, randomized, crossover design was used [17]. All the participants completed 1 pretrial and 3 main trials in 1 month. To prevent a carry-over effect and the effect of individual differences in recovery, washout periods of at least 72 h were arranged [2]. On the trial day, participants were refrained from ingesting any food or drink with caffeine, alcohol, or nicotine. In addition, they had restrictions on performing heavy exercises on the day before the main trial. For the main trials, participants were allocated to different small groups based on their maximal oxygen consumption (VO_2max_), i.e., participants with similar VO_2max_ were assigned to the same group. Each participant was randomized to a sequence of three treatments (i.e., Control; CHO; CHO_M) by using Excel (Microsoft, Redmond, WA, USA).

#### 2.2.1. Pretrial

To estimate VO_2max_ in the pretrial, the participants were instructed to finish a 20 m beep test (starting at 8.0 km/h and increasing by 0.5 km/h for each one-minute stage) after a warm-up session [18]. The warm-up protocol involved running for five laps around the field (i.e., 600 m) with low to moderate intensity, both static and dynamic stretching, 20 m of running at 55% VO_2max_, 20 m of running at 95% VO_2max_, and vertical jump (as high as possible) for three times. Participants could change warm-up intensity and time based on their personal habits, as long as it was finished within ten minutes. After that, participants had their body height and weight measured, and a detailed demographic information form completed. Furthermore, participants read the hard copy of the experimental protocol of the main trial to familiarize themselves with the study procedures.

#### 2.2.2. Main Trial

The main trials were conducted during the daytime (i.e., 9:00 a.m.–5:30 p.m.). The average temperature was 28.9 ± 5.2 °C and average air humidity was 75.7% ± 5.8%. Two hours before entering the test field, participants were instructed to drink 500 mL of plain water to be normally hydrated [19]. Bladders were to be completely emptied before the main trials

The Loughborough Intermittent Shuttle Test [20] was selected, given that it is a reliable action mode to simulate athletic performance in soccer matches. The protocol consists of six 15-min blocks of exercise separated by 3-min rest periods. The exercises of each block include 10–12 cycles of different activities (i.e., a 20-min walk, 20-min maximal sprint, 4-s standing rest, 20-min run at approximately 55% VO_2max_ pace, and 20-min run at approximately 95% VO_2max_ pace). In the main trials, participants were instructed to finish the first 3 blocks of protocol to simulate the exercise intensity of the first half of a soccer match, and to finish the Stroop test on screen during the interval of each block to simulate the cognitive consumption of playing a soccer match. [21].

Participants’ blood samples in the pretest were collected after they arrived at the test field. Then they were instructed to complete a 10-min warm-up following a standard protocol as described in pretrial. Subsequently, the warm-up vertical jump, 20-m sprint, rating of perceived exertion (RPE), muscle pain, and mental fatigue were measured. During the break between two blocks, participants were asked to report their RPE level (i.e., second and third tests) and complete one Stroop test to induce mental fatigue.

**Figure 1:**
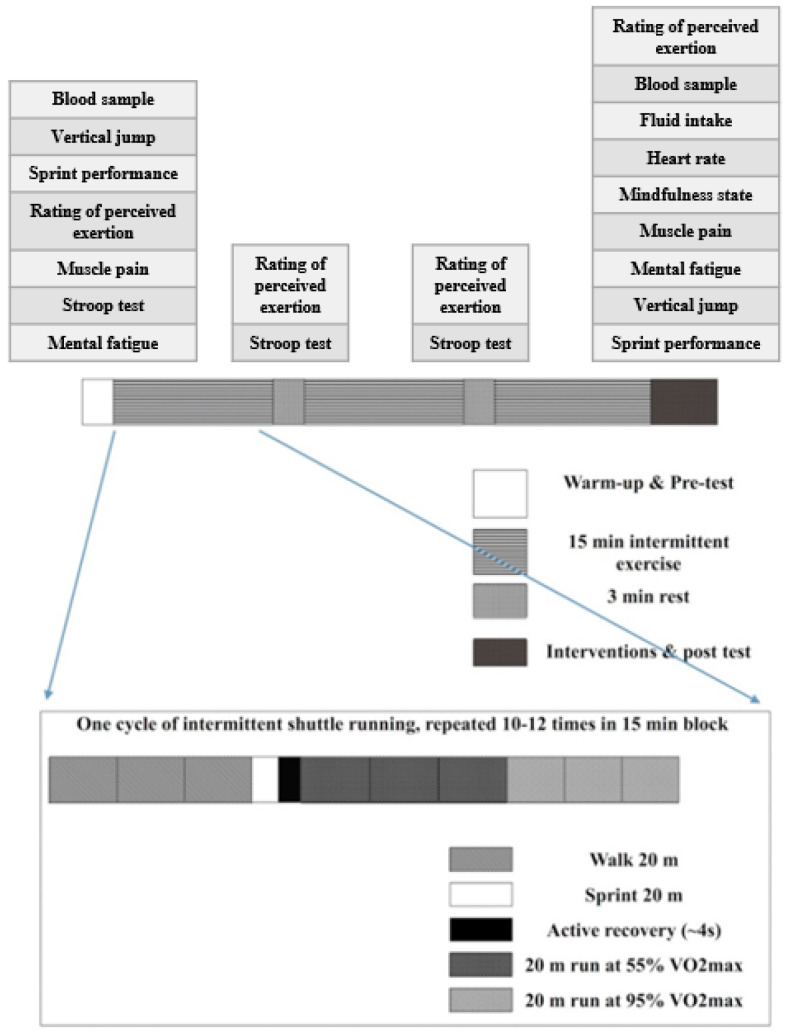
Adopted from the Loughborough Intermittent Shuttle Test.

After finishing the first 3 blocks of exercise protocol, all participants had a half-time break that simulated the half-time break of a real soccer match. At the start of half-time, participants were asked to report their RPE (i.e., fourth test), provide a blood sample (i.e., mid-test), and drink a beverage. There were 2 different beverages (i.e., with and without CHO) contained in unmarked paper cups. Then they were required to listen to a 6-min audio labelled with numbers followed by the report of mindfulness level, muscle pain and mental fatigue. To avoid injury and maintain sport performance, they were instructed to do a standardized warm-up for 3 min (i.e., 200 m running, dynamic stretches, a 90% VO_2max_ sprint for 50 m and 2 vertical jumps) before the posttest for vertical jump and sprint performance. The processes of post tests were the same as those of the pretest except that the sprint performance test was repeated 6 times with a 30-s interval. Finally, participants’ RPE (posttest) and blood samples (i.e., posttest) were collected. The protocol is illustrated in Figure 1.

### 2.3. Treatment

In the half-time break, three different treatments (i.e., CHO, CHO_M, and Control, as defined below) were applied.

Treatment CHO involved a CHO-electrolyte solution (Aquarius^®^, Coca-Cola Co., Hong Kong, China), contained with 4.2 g CHO, 30 mg sodium, 7 mg potassium, 0.7 mg calcium, 1.1 mg magnesium, and 18 kcal energy, 3 mL/kg of which was to be consumed by the participants in each trial [22]. In addition, the researchers instructed them to listen to a 6-min travelling introduction, an audio recording of a Chinese tourist attraction, without mindfulness content, as an active control.

Treatment CHO_M involved the same CHO-electrolyte solution as well as a 6-min MBI comprising mindful breathing and body scanning.

The Control treatment involved the same electrolyte solution but without CHO and energy (Aquarius Zero^®^, Coca-Cola Co., Hong Kong, China), with the same volume, time, and steps, as stated in CHO treatment.

### 2.4. Measurements

#### 2.4.1. Heart Rate

The researchers recorded the heart rate (HR) during the half-time using an HR monitor (Polar H10, Polar Electro Oy, Kempele, Finland) and an iPad (2018 version, Apple Inc., Cupertino, CA, USA).

#### 2.4.2. Blood Samples

Blood glucose was measured using a portable glucose analyzer (Accu-Chek Performa Nano, Roche, Germany). Blood lactate was assessed using a handheld portable lactate analyzer (Lactate Plus, Nova Biomedical, Waltham, MA, USA).

#### 2.4.3. Vertical Jump

Vertical jump performance (i.e., jump height, flight time, velocity, force, and power) was assessed through the application “My Jump II” on the iPad (2018 version, Apple Inc., Cupertino, CA, USA). Jump height is determined by the app using the equation *h* = *t*^2^ × 1.22625, described by Bosco et al., where *h* stands for the jump height and *t* for flight time [23]. All collections were made with the same iPad and by the same researcher with no professional experience in video analysis. The researcher was always recording from the same position and with the same distance from the participants (i.e., 1.5 m). The validity and reliability of the application has previously been proven [24].

#### 2.4.4. Sprint Performance

The 20-m sprint performance was measured using the Kinematic Measurement System ((KMS) Innervation, Perth, Australia), a visible red-light system modulated by a single beam with 2 sets of gates and polarizing filters. The time between the points when the participants passed through the first and second gates was recorded as the sprint performance.

#### 2.4.5. Mindfulness State

Similar to other studies [17], the researchers asked the participants to answer 2 statements (“I felt in touch with my body” and “I focused on my breathing”) as a manipulation check of mindfulness induction immediately after completing the treatments. A 7-point Likert scale, ranging from 0 (very slightly or not at all) to 6 (extremely), was used for responses. A higher mindfulness score by averaging the two responses indicated a higher mindfulness level.

#### 2.4.6. Muscle Pain

Levels of muscle pain were evaluated by a pain intensity scale ranging from 0 (no pain at all) to 10 (extremely unbearable), as used in previous research [25].

#### 2.4.7. Perceived Exertion

The RPE was assessed using the Borg 15-point RPE scale [26], ranging from 6 (very light) to 20 (extremely unbearable).

### 2.5. Statistical Analysis

A normality test was assessed for all variables. ANOVAs were performed, as the F-test remains a valid statistical procedure under non-normality in a variety of conditions [27,28]. A two-way repeated measures analysis of variance (ANOVA) was used to detect the main and interactive effects of treatments (i.e., Control, CHO, and CHO_M) and time (i.e., pretest and posttest) on participants’ performance (i.e., vertical jump, sprint performance, RPE level, blood glucose and lactate, muscle pain, and mental fatigue). A one-way repeated ANOVA was used to test the difference of mindfulness state among three groups. For variables that adopted multiple posttests (i.e., sprint), the difference between pretest and each posttest was computed for further analysis. If a significant effect was observed, post hoc tests with Bonferroni correction were conducted. The significance level was set at α = 0.05, and effect size was referred to by ŋ^2^ (partial eta squared). The analyses were conducted in SPSS 25.0 (SPSS, Inc., Chicago, IL, USA).

## 3. Results

### 3.1. Vertical Jump

Table 1 presents the mean (M) and standard deviation (SD) of five indices of vertical jump (i.e., height, flight time, velocity, force, and power). The five indices did not exhibit any interactive and treatment effects. Three indices exhibited significant time differences, with the values of pretest being higher than those of posttest (*p* < 0.01, partial ŋ^2^ = 0.65 for flight time; *p* < 0.01, partial ŋ^2^ = 0.65 for velocity; and *p* < 0.01, partial ŋ^2^ = 0.67 for height).

### 3.2. Sprint Performance

#### 3.2.1. Sprint Performance—Difference between Pretest and Each of the Posttests

Table 2 presents the difference of sprint performance between pretest and each of six posttests. A significant interactive effect (*p* = 0.02, partial ŋ^2^ = 0.20) and treatment effect (*p* = 0.01, partial ŋ^2^ = 0.30) was observed. Post hoc analysis revealed that the CHO_M group performed better than the CHO (*p* = 0.02) and Control groups (*p* = 0.02) in post repeated sprint tests. No group differences were detected between CHO and Control groups (*p* = 1.00) (Table 2).

#### 3.2.2. Sprint Performance—Pretest and First Posttest

No significant interaction effect on sprint performance (*p* = 0.12, partial ŋ^2^ = 0.30) was found. There was no significant treatment effect on sprint performance (*p* = 0.60, partial ŋ^2^ = 0.08), but time had a significant main effect (*p* < 0.01, partial ŋ^2^ = 0.50). Post hoc analysis revealed that sprint performance in the pretest (M = 3.32, SD = 0.03) was significantly better than that in the first posttest (M = 3.42, SD = 0.05; *p* < 0.01).

### 3.3. Mindfulness State

The mindfulness state of the three groups showed significant differences (*p* < 0.01, partial η^2^ = 0.97). The post hoc tests showed that participants in the CHO_M group had significantly higher mindfulness levels (M = 5.32; SD = 0.15) than those in the Control (M = 2.11; SD = 0.19; *p* < 0.01) and CHO groups (M = 2.43; SD = 0.27; *p* < 0.01). However, the Control and CHO groups showed no significant difference (*p* = 1.00).

### 3.4. Blood Glucose and Lactate

The interaction effect was not statistically significant in terms of blood glucose (*p* = 0.60, partial ŋ^2^ = 0.22). The treatment had no significant effect (*p* = 0.86, partial ŋ^2^ = 0.03), but there was significant time effect on blood glucose (*p* < 0.01, partial ŋ^2^ = 0.60). Post hoc analysis revealed that the mid-test glucose level (M = 6.61, SD = 0.21) was significantly higher than that of the posttest (M = 5.74, SD = 0.11; *p* < 0.01).

For lactate, there was no interactive effect (*p* = 0.31, partial ŋ^2^ = 0.09). A significant main effect was revealed in terms of time (*p* < 0.01, partial ŋ^2^ = 0.96) but not on treatment (*p* = 0.15, partial ŋ^2^ = 0.27). The post hoc test showed that the mid-test lactate level (M = 6.06, SD = 0.33) was significantly higher than that of the pretest (M = 1.58, SD = 0.16; *p* < 0.01) and posttest (M = 5.82, SD = 0.29; *p* < 0.01). Moreover, the posttest lactate level was higher than that of the pretest (*p* < 0.01).

### 3.5. Muscle Pain and Mental Fatigue

The interaction effect between treatment and time was not statistically significant in terms of muscle pain (*p* = 0.29, partial ŋ^2^ = 0.19). There was no significant treatment effect (*p* = 0.51, partial ŋ^2^ = 0.11), but time had a significant main effect (*p* < 0.01, partial ŋ^2^ = 0.89). Post hoc analysis revealed that the muscle pain in the pretest (M = 1.93, SD = 0.24) was significantly lower than that in the posttest (M = 4.76, SD = 0.26; *p* < 0.01).

**Figure 2:**
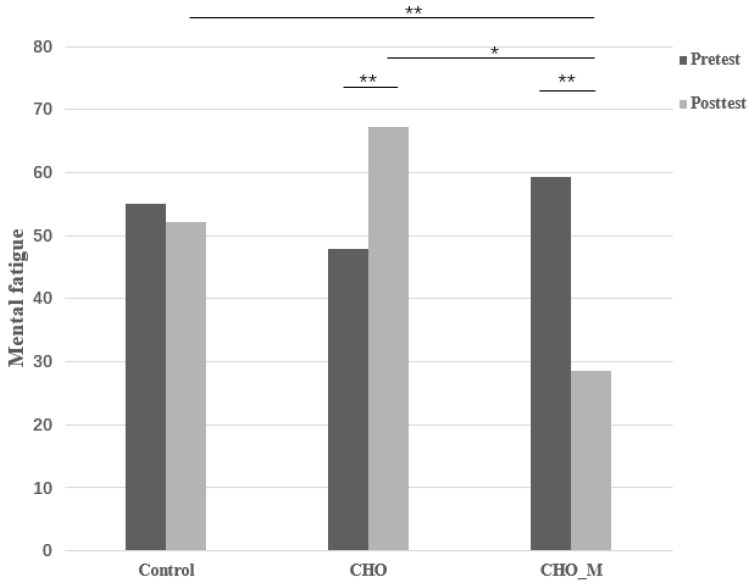
Changes in the 3 groups’ mental fatigue through time. * *p* = 0.05; ** *p* < 0.01.

Regarding mental fatigue, a significant interactive effect was observed (*p* < 0.01, partial ŋ^2^ = 0.65). Post hoc analysis showed that there was a significant treatment effect in posttest (Control vs. CHO vs. CHO_M: 52.14 ± 7.43 vs. 67.14 ± 4.25 vs. 28.57 ± 6.10; *p* = 0.23 for Control vs. CHO; *p* = 0.05 for CHO vs. CHO_M and *p* < 0.01 for Control vs. CHO_M). A significant within group difference was also observed in both CHO (pre vs. post: 47.86 ± 23.92 vs. 67.14 ± 15.90, *p* < 0.01) and CHO_M groups (pre vs. post: 59.29 ± 15.92 vs. 28.57 ± 22.82, *p* < 0.01) but not in the control group (pre vs. post: 55.00 ± 14.01 vs. 52.14 ± 27.78, *p* = 0.68) (Figure 2).

### 3.6. Rate of Perceived Exertion

The interaction was not significant (*p* = 0.44, partial ŋ^2^ = 0.07). No significant treatment effect was detected (*p* = 0.77, partial ŋ^2^ = 0.04), but the main effect of time was significant (*p* < 0.01, partial ŋ^2^ = 0.95). According to the post hoc analysis, the pretest scores (M = 8.33, SD = 0.42) were significantly lower than other following scores. Scores of the third (M = 18.69, SD = 0.41) and fourth tests (M = 19.31, SD = 0.23) were the two highest, followed by the second (M = 16.244, SD = 0.41) and posttest (M = 16.17, SD = 0.27). No difference was found between the third and mid-tests and between the second and posttest.

### 3.7. Heart Rate

Results indicated that no interactive effect was detected (*p* = 0.26, partial ŋ^2^ = 0.09). There was no significant treatment effect (*p* = 0.24, partial ŋ^2^ = 0.20), but a significant time effect (*p* < 0.01, partial ŋ^2^ = 0.73). Post hoc analysis showed that participants’ heartrate decreased significantly during the intervention period (pre vs. post: M = 129.14, SD = 11.27; M = 115.19, SD = 11.71, *p* < 0.01).

## 4. Discussion

To our knowledge, this study is the first field trial that investigated the application of brief MBI coupled with CHO on soccer players’ recovery during a half-time break. Applying brief MBI coupled with CHO ingestion during half-time breaks was found to significantly increase athletes’ mindfulness levels and decrease athletes’ mental fatigue. In addition, compared with the participants in Control and CHO groups, participants in the CHO_M performed better in the repeated sprint tests but not in single measures (e.g., vertical jump, pretest, and first posttest in sprint performance).

As hypothesized, participants who received brief MBI coupled with CHO during the half-time break performed better in some facets when compared with those who received other treatments (e.g., sprint performance). Brief MBI was also helpful for recovery from mental fatigue. As previous literature has demonstrated that both acceptant mood and relaxing breath positively affect the brain, the capacity of mindfulness to improve acceptance and relaxation can affect the biological system that regulates the generation of mental fatigue [29]. With regard to our observations about mental fatigue, three potential mechanisms were raised. First, as Smith [30] pointed out in a review study, the causation of mental fatigue during a soccer games is influenced by the anterior cingulate cortex (i.e., it increases adenosine and decreases dopamine). Given that previous studies have demonstrated stronger subgenual and adjacent ventral anterior cingulate cortex activity, which controls parasympathetic activity, in the MBI condition through imaging data [31], it is possible to restore mental fatigue through MBI. Second, MBI has been proven to improve respiratory sinus arrhythmia [32], a variation of heart rate in synchrony with respiration [33] that increases in the resting state and decreases in conditions of stress or tension. Therefore, possibly, the increase of respiratory sinus arrhythmia is closer to the resting state and thus relieves mental fatigue. Third, respiratory sinus arrhythmia is a valid and reliable biomarker of emotional regulation capacity in humans, and has been frequently used as a noninvasive method for investigating cardiac vagal tone [34,35]. In addition, mindfulness is beneficial to the prefrontal cortex, which can modulate brain activities in multiple emotion-processing systems [36]. Therefore, mindfulness has potential effects on emotional regulation and mental fatigue.

One explanation for the observed improvement in repetitive sprinting is that, in general, the sympathetic nervous system (SNS) and parasympathetic nervous system (PNS) are in equilibrium in the rest state. During exercise, increased levels of SNS activity upsets the balance, leading to biological changes such as pupil dilation, faster heart beats, atelectasis, and blood pressure elevation [37]. After receiving the acceptance hint (i.e., MBI), the activity levels of the PNS are induced, thus promoting the “rest and digest” response that calms the body down [16]. Another reason for the observed improvement in repeated sprinting is that it might be affected by cerebral oxygenation, which is related to the prefrontal cortex [38]. Previous fMRI studies have determined that MBI regulates the activity of the prefrontal cortex [36]. Therefore, we observed an increase in repeated sprinting performance. For vertical jump, three of the five indices exhibited significant pre–post differences, while the other two did not. This is not very surprising, as a previous review study has indicated that compared with other indices, power should be reported with caution [39]. Additionally, one possible explanation is that after half time break, athletes’ power and force recovered relatively faster than the other three indices.

Although some previous studies have confirmed the immediate effect of MBI on releasing pathological pain [13], the same results were not observed in the present research. Probably, short-term mindfulness training for acute pain is not always effective [40]. This is because the mechanisms that cause pathological pain and those that cause exercise-induced muscle pain are different from each other [41,42]. As the improved immune system is probably the mediator between MBI and pathological pain [11], such a link may be absent in sports-related muscle damage. Another potential reason might be that there was no effect of mindfulness on adjusting endorphin levels, which can cause muscle pain [43].

Further, although previous studies have asserted that MBI positively affects HR recovery by improving cardiac efficiency [14], no statistically significant group difference was observed in our research. This is probably due to participants in this study being beginners in the use of mindfulness techniques. Another possible reason is that the period of receiving MBI in this study was also the period of a transition from high-intensity exercise to a relaxed state. Therefore, the heart rate changes due to the difference of activity state may buffer the change caused by MBI. Further research on this phenomenon is necessary.

No statistically significant influence was observed on the values of blood glucose and lactate between the Control, CHO, and CHO_M groups. This result is consistent with previous studies in which both ingestion of a CHO beverage before and during a competition had no effect on participants’ blood glucose [44]. A possible explanation is that respiratory sinus arrhythmia may have evolved to save energy for both cardiac and respiratory systems by suppressing unnecessary heartbeats during exhalation and ineffective ventilation during the ebb of perfusion (delivery of blood from arteries to capillaries for oxygenation and nutrition) [33,45]. Therefore, MBI practice may delay the effect of CHO digestion and glucose absorption at the capillaries during the test.

Our findings also indicate that the ingestion of CHO during a half-time break may have both positive and negative effects on the initial stage of second-half performance. Although previous review studies have reported that the ingestion of CHO in prolonged exercise can enhance athletes’ performance and maintain their endurance [6], this study found CHO to have a significant negative effect on mental fatigue. This is in line with a previous meta-analysis that concluded that CHO ingestion does not have a beneficial effect on mood and may increase fatigue within 30 min post-consumption [46]. In reference to the mentioned results, MBI seems to have the effect of buffering the negative influence of CHO. This point also requires further investigation.

The results of our study have practical implications. It has been confirmed that MBI can be used during half-time breaks of soccer games. Although mindfulness resulted in a significant increase in RSA compared with a relaxation strategy in a nonathletic- or nonexercise-population [32], considering that half-time is short and important, MBI’s superiority to other mainstream psychological interventions (e.g., self-talk, goal setting, imagery) needs to be further investigated. The current study was a field test that could simulate exercise intensity but not athletes’ moods during the game. Future studies should be conducted in real games.

This study has several limitations. First, although the current trial simulated the exercise intensity of half of a soccer match, the atmosphere of competition was not simulated. As previous studies have suggested, athletes’ moods could affect their performance [47], and it is possible that the atmosphere of real competition may affect the physiological and psychological measurements in the current study. Future studies may consider conducting trials in a real soccer competition. Second, although ingesting CHO is a convenient and common recovery strategy during the half-time break, no control group with MBI alone was involved in the current study. Future research may consider investigating the unique effect of brief MBI alone on soccer players.

## 5. Conclusions

In conclusion, our field experiment provides preliminary evidence of the positive effects of brief MBI coupled with ingestion of CHO on athletes’ recovery from fatigue in the initial stage of the second half of a game.

## Figures and Tables

**Table 1:** The performance of five dimensions for vertical jump between pre and post-test (M ± SD).

Variable	Pre			Post		
	Control	CHO	CHO_M	Control	CHO	CHO_M
Height (cm)	42.92	42.08	43.27	39.45	40.32	41.84
	(6.93)	(8.10)	(8.08)	(7.48)	(8.15)	(8.28)
Flight time (ms)	589.93	583.43	591.71	564.86	570.93	581.64
	(46.81)	(55.51)	(54.41)	(53.42)	(56.72)	(56.68)
Velocity (m/s)	1.45	1.43	1.45	1.39	1.40	1.43
	(0.11)	(0.14)	(0.13)	(0.13)	(0.14)	(0.14)
Force (N)	1308.92	1365.90	1360.36	1294.10	1376.68	1356.29
	(139.02)	(232.93)	(179.85)	(188.70)	(213.91)	(177.09)
Power (W)	1905.58	1976.70	1988.99	1809.15	1945.52	1941.32
	(337.06)	(500.84)	(405.81)	(396.63)	(454.65)	(413.26)

**Table 2:** The sprint performance—difference between pretest and each of the posttests (M ± SD).

	Post					
	1	2	3	4	5	6
Control	0.10 (0.20)	0.10 (0.26)	0.15 (0.16)	0.19 (0.24)	0.48 (0.14)	0.11 (0.12)
CHO	0.15 (0.20)	0.15 (0.22)	0.13 (0.14)	0.10 (0.17)	0.85 (0.12)	0.07 (0.15)
CHO_M ^**a,b**^	0.14 (0.19)	0.06 (0.23)	0.16 (0.15)	0.05 (0.19)	0.07 (0.12)	−0.01 (0.15)

**^a^***p* < 0.05, CHO_M vs. Control; **^b^** *p* < 0.05, CHO_M vs. CHO.
